# A population-based analysis of spirometry use and the prevalence of chronic obstructive pulmonary disease in lung cancer

**DOI:** 10.1186/s12885-020-07719-y

**Published:** 2021-01-05

**Authors:** Sophie Corriveau, Gregory R. Pond, Grace H. Tang, John R. Goffin

**Affiliations:** 1 Division of Respirology, Department of Medicine, McMaster University, Hamilton, Ontario Canada; 2 Department of Oncology, McMaster University, Hamilton, Ontario Canada; 3 Department of Health Research Methods, Evidence, and Impact, Faculty of Health Sciences, McMaster University, Hamilton, Ontario Canada; 4 Division of Medical Oncology, Department of Oncology, McMaster University, Hamilton, Ontario Canada

**Keywords:** COPD, Lung cancer, Spirometry

## Abstract

**Background:**

Chronic obstructive pulmonary disease (COPD) and lung cancer are associated diseases. COPD is underdiagnosed and thus undertreated, but there is limited data on COPD diagnosis in the setting of lung cancer. We assessed the diagnosis of COPD with lung cancer in a large public healthcare system.

**Methods:**

Anonymous administrative data was acquired from ICES, which links demographics, hospital records, physician billing, and cancer registry data in Ontario, Canada. Individuals age 35 or older with COPD were identified through a validated, ICES-derived cohort and spirometry use was derived from physician billings. Statistical comparisons were made using Wilcoxon rank sum, Cochran-Armitage, and chi-square tests.

**Results:**

From 2002 to 2014, 756,786 individuals were diagnosed with COPD, with a 2014 prevalence of 9.3%. Of these, 51.9% never underwent spirometry. During the same period, 105,304 individuals were diagnosed with lung cancer, among whom COPD was previously diagnosed in 34.9%. Having COPD prior to lung cancer was associated with lower income, a rural dwelling, a lower Charlson morbidity score, and less frequent stage IV disease (48 vs 54%, *p* < 0.001). Spirometry was more commonly undertaken in early stage disease (90.6% in stage I-II vs. 54.4% in stage III-IV).

**Conclusion:**

Over a third of individuals with lung cancer had a prior diagnosis of COPD. Among individuals with advanced lung cancer, greater use of spirometry and diagnosis of COPD may help to mitigate respiratory symptoms.

## Background

Chronic obstructive pulmonary disease (COPD) is a major cause for morbidity and is currently the 4th leading cause of death worldwide, with an estimated prevalence of nearly 11% among those age 30 or more [1]. Worldwide, tobacco smoke is the most common risk factor for COPD [2]. COPD is characterized by persistent inflammation and remodelling of the airways, pulmonary parenchyma, and vasculature, leading to airflow obstruction [3].

Among cancers, lung cancer remains the leading cause of death among both men and women in Canada [4]. Lung cancer and COPD are interrelated diseases sharing commonalities of tobacco use and chronic inflammation [5]. Among lung cancer patients, a COPD prevalence of 22–52% has been reported [6–8] Epidemiological data show that COPD is a major independent risk factor for lung cancer even after adjusting for smoking, conferring a 2-fold increased risk [5, 8].

Dyspnea can be a debilitating symptom and is common in both COPD and lung cancer [9]. While treatments are available for COPD, underdiagnosis necessarily leads to undertreatment [7]. While most studies are directed toward patients with at least moderate lung dysfunction, studies in COPD have shown that inhaled agents can improve symptoms and lung function, modestly improve quality of life, and reduce COPD exacerbations [10]. Oral corticosteroids can decrease hospitalization during COPD exacerbations, while oxygen use for hypoxemia improves mortality [10]. Conversely, while some conditions in lung cancer may be reversible, tools for the general treatment of dyspnea are limited, and most studies have been undertaken near the end of life [11, 12].

Given the improved outcomes expected amongst patients with lung cancer due to novel treatments [13], and the known relationship between COPD and lung cancer, the burden due to COPD amongst patients with lung cancer is likely to increase. While previous studies have shown a high prevalence of COPD among lung cancer patients in institutional or survey-based studies [6–8], there is a paucity of population-based data. Furthermore, as spirometry is the gold standard for COPD diagnosis, it is important to understand the use of spirometry in the lung cancer population. Therefore, this study used a population-based administrative dataset in Ontario, Canada, to assess both the prevalence of COPD in lung cancer in a real-world setting as well as the use of spirometry for appropriate diagnosis.

## Methods

### ICES database

Data was obtained from linked administrative databases created by ICES, a provincially funded research organization that provides a secure and accessible array of the province of Ontario’s demographic and health-related data. All residents of Ontario have a universal public health insurance plan and therefore all medically necessary data for residents, providers and hospitals can be captured. ICES provides anonymized access to demographic data, cancer registry data, ambulatory and hospital records, physician billing data, and vital statistics, all linked at the patient level. All administrative data sets are overlapping subsets of the Ontario population. Using the 2014 Statistics Canada estimate, the Ontario population was 13.6 million, being 50.8% female, and with 7.8 million being of age 35 years and older (57%) [14].

Persons with COPD were identified from the validated ICES-derived cohort available from 1996 to 2014 [14]. This cohort definition is based on finding at least one Ontario physician claim or hospitalization for COPD among individuals 35 years and older. This definition was validated by Gershon and colleagues through expert consensus analysis of 442 primary care charts as compared with health administration data [15]. The chart standard used all available information but did not require spirometry. The administrative cohort definition has a sensitivity of 85% and a specificity of 78% when compared with actual patient chart data.

### COPD amongst lung cancer patients

The population of interest was all individuals in Ontario diagnosed with lung cancer as identified by the Cancer Care Ontario cancer registry database. Patients with no follow-up information post-diagnosis, or where last follow-up/death date occurred prior to diagnosis (indicative of autopsy diagnosis or a data error) were excluded.

Patient variables collected included sex, income quintile, rural status, John Hopkins Adjusted Clinical Groups resource utilization band (RUB), and Charlson comorbidity score. Dwelling status was defined by Statistics Canada as rural if the community size is less than 10,000, and is dichotomous, with all others being considered urban. The RUB is a part of John Hopkins Adjusted Clinical Groups case mix system, which is used to estimate an individual’s insurance needs. The system groups medical diagnoses into Aggregated Diagnosis Groups (ADG’s) according to expected severity, duration, diagnostic uncertainty, etiology (e.g. infectious, neoplastic), and likelihood of requiring specialist care or hospitalization. The ADG defines a level of expected healthcare needs, and different diseases can fall into the same ADG based on the above factors. The RUB groups individuals by demographics and ADG to assign 6 levels of risk for requiring medical resources (bands 0–5). A higher band level indicates a higher risk. The Charlson Comorbidity Index is a method of categorizing comorbidities of patients based on the International Classification of Diseases (ICD) diagnosis codes. Each comorbidity category has an associated weight (from 1 to 6), based on the adjusted risk of mortality or resource use, and the sum of all the weight results in a single comorbidity score for a patient. A score of zero for instance, indicates that no comorbidities were found. The higher the score, the more likely the predicted outcome will result in mortality or high resource use. Both the Charlson score and RUB are based upon administrative data during the 2 years prior to the index date, that being the first instance of a COPD or lung cancer diagnosis.

### COPD prevalence in Ontario

Prevalence of COPD in Ontario was calculated as of 2014, by determining the total number of individuals diagnosed with COPD between 1996 and 2014 who were alive at the data cutoff date (31 December 2014), divided by the Statistics Canada [16] estimated population of Ontario. Incidence and prevalence rates were then reported per 100,000 individuals.

### Spirometry and cancer

Separately for our study, spirometry use was derived from physician billing data. In the fee-for service setting of Ontario, we expect the billing capture of spirometry to have good fidelity, as it provides both technical and professional reimbursement. Lung cancer and staging information was defined using ICD-9 and ICD-10 codes and the Ontario Cancer Registry, capturing all diagnoses in Ontario, Canada, from 1994 to 2014. All data were merged and de-identified using standard ICES policies, such as providing age in 5-year age groupings (e.g. 35–39, 40–44, etc) and replacing specific dates by anchoring the dates to the first diagnosis (COPD or lung cancer) date. Data cutoff for this analysis was 31 December 2014.

### Statistical analysis

Analysis was conducted using ICES’s confidential, analytic, virtual environment using SAS v9.3. Descriptive statistics (mean, median, standard deviation, interquartile range, proportions) was used to describe demographic data. Lung cancer patients were dichotomized into patients who had a diagnosis of COPD prior to the day of lung cancer diagnosis versus those with no known diagnosis of COPD. Statistical tests were performed comparing patient factors between these two groups of patients using the Wilcoxon rank sum test (for age based on ordinal groups), the Cochran-Armitage test (for income quintile, RUB and stage) and the chi-square test (for sex, rurality and Charlson score). All tests were two-sided and a *P*-value of 0.05 or less was considered statistically significant. The local ethics board approved the study.

## Results

### Lung cancer

**Fig. 1:**
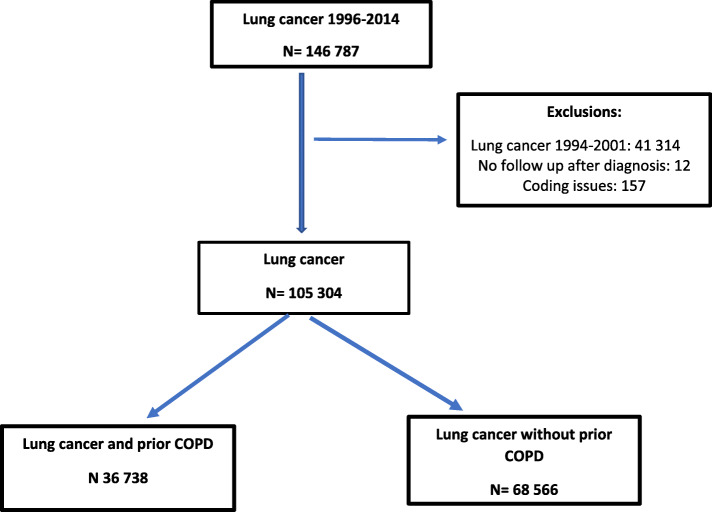
Consort diagram flow chart for lung cancer patients

Between 1996 and 2014, 146,787 Ontarians were diagnosed with lung cancer. After exclusions, 105,304 patients were included in this analysis (Fig. 1). Just under half (49453) were female and median age of patients was between 65 and 69 years at diagnosis. Stage information was only available on 43,375, however, of these patients, 22,507 (51.9%) were stage 4. Demographic information is available in Table 1.

**Table 1:** Ontario lung cancer patient demographics and clinical characteristics among those with and without a prior diagnosis of COPD

Characteristic	Statistic	N	All Patients	Prior COPD	No Prior COPD
N			105,304	36,738	68,566
Sex	N (%) Female	105,304	49,453 (47.0)	17,127 (46.6)	32,326 (47.2)
Age Group	≤39	105,304	882 (0.8)	235 (0.6)	647 (0.9)*
	40–44		1594 (1.5)	673 (1.8)	921 (1.3)
	45–49		3746 (3.6)	1563 (4.3)	2183 (3.2)
	50–54		6973 (6.6)	2793 (7.6)	4180 (6.1)
	55–59		11,099 (10.5)	4574 (12.5)	6525 (9.5)
	60–64		14,907 (14.2)	5999 (16.3)	8908 (13.0)
	65–69		17,347 (16.5)	6985 (19.0)	10,362 (15.1)
	70–74		17,213 (16.4)	6152 (16.8)	11,061 (16.1)
	75–79		14,959 (14.2)	4419 (12.0)	10,540 (15.4)
	80–84		10,111 (9.6)	2301 (6.3)	7810 (11.4)
	85+		6473 (6.2)	1044 (2.8)	5429 (7.9)
Income Quintile	N (%) Lowest	104,843	24,522 (23.4)	9131 (25.0)	15,391 (22.6)*
	2		23,327 (22.3)	8493 (23.2)	14,834 (21.7)
	3		20,506 (19.6)	7199 (19.7)	13,307 (19.5)
	4		19,211 (18.3)	6258 (17.1)	12,953 (19.0)
	Highest		17,277 (16.5)	5520 (15.1)	11,757 (17.2)
Rural	N (%) Yes	105,189	17,188 (16.3)	6710 (18.2)	10,593 (15.3)*
Charlson Score	N (%) ≥1	105,304	13,503 (12.8)	3415 (9.3)	10,088 (14.7)*
Resource Utilization Band	0	105,304	2186 (2.1)	952 (2.6)	1234 (1.8)*
	1		1717 (1.6)	940 (2.6)	777 (1.1)
	2		5150 (4.9)	2674 (7.3)	2476 (3.6)
	3		45,558 (43.3)	18,727 (51.0)	26,831 (39.1)
	4		28,314 (26.9)	8269 (22.5)	20,045 (29.2)
	5		22,379 (21.3)	5176 (14.1)	17,203 (25.1)
Lung cancer stage	N (%) 1	43,375	8639 (19.9)	3607 (21.2)	5032 (19.1)*
	2		3422 (7.9)	1464 (8.6)	1958 (7.4)
	3		8807 (20.3)	3717 (21.9)	5090 (19.3)
	4		22,507 (51.9)	8205 (48.3)	14,302 (54.2)

*means statistically significant (*p* < 0.001)

At the time of their lung cancer diagnosis, 36,738 (34.9%) had COPD diagnosed on the same day or previously, with 16.8% having a diagnosis of COPD within 5 years preceding their lung cancer diagnosis. Of these patients, one-quarter (9416) never underwent spirometry testing, and 63.5% (23321) had spirometry testing prior to their lung cancer diagnosis (see Table 2).

**Table 2:** The proportion of spirometry use in patients with lung CA and dx of COPD on day of lung CA dx by year

Year	Total Lung CA Patients	Lung CA Patients with Known COPD on day of Dx	No Spirometry	Spirometry Prior to Lung CA Dx	Spirometry Post Lung CA Dx
2002	7159	1948	671 (34.5)	1079 (55.4)	198 (10.2)
2003	7143	2020	683 (33.8)	1141 (56.5)	196 (9.7)
2004	7381	2208	723 (32.7)	1253 (56.8)	232 (10.5)
2005	7821	2450	696 (28.4)	1451 (59.2)	303 (12.4)
2006	7841	2539	698 (27.5)	1581 (62.3)	260 (10.2)
2007	7851	2714	735 (27.1)	1634 (60.2)	345 (12.7)
2008	7925	2753	672 (24.4)	1781 (64.7)	300 (10.9)
2009	8098	3072	769 (25.0)	1977 (64.4)	326 (10.6)
2010	9066	3395	812 (23.9)	2227 (65.6)	356 (10.5)
2011	9122	3537	820 (23.2)	2326 (65.8)	391 (11.1)
2012	9371	3663	809 (22.1)	2481 (67.7)	373 (10.2)
2013	8760	3531	785 (22.2)	2363 (66.9)	383 (10.9)
2014	7766	2908	543 (18.7)	2027 (69.7)	338 (11.6)
Total	105,304	36,738	9416 (25.6)	23,321 (63.5)	4001 (10.9)

For individuals with registry recorded stage data, 12,061 persons had stage I-II lung cancer, of whom 10,931 (90.6%) had spirometry at some point. Conversely, among 31,314 persons with stage III-IV lung cancer, 17,041 (54.4%) of patients had spirometry (*p* < 0.01 early vs late stage).

In contrast, of 68,566 lung cancer patients who did not have COPD diagnosed at the time of their lung cancer diagnosis, 32,889 (48.0%) never underwent spirometry testing, while 20,755 (30.3%) had prior spirometry.

Small but statistically significant differences were seen between those with and without a COPD diagnosis prior to a diagnosis of lung cancer. Those with a COPD diagnosis were younger, of lower income, more commonly dwelt in rural areas, had a lower Charlson score and RUB, and less likely to be stage IV (48 vs 54%) (*p* < 0.001 for each) (see Table 1).

### COPD

**Fig. 2:**
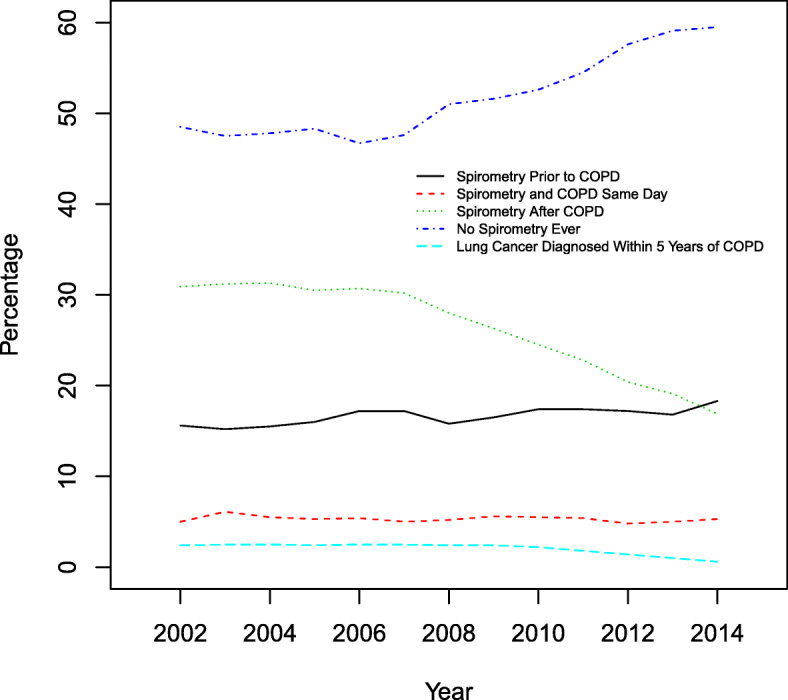
The frequency of spirometry use, by incidence of COPD by year, from 2002 to 2014, and the frequency of lung cancer diagnosed within 5 years of COPD

By 2014, it is estimated there were a total of 722,896 Ontarians (364,196 females and 358,700 males) living with COPD, a prevalence rate of 926/100000 (898/100000 females and 957/100000 males). Of the 756,786 individuals diagnosed with COPD from 2002 to 2014, just over half (393096) had never undergone spirometry testing. The majority of individuals have spirometry testing after the diagnosis of COPD is made (78%) (Fig. 2).

Among individuals with a diagnosis of COPD, 2.4 to 2.5% of individuals were diagnosed with lung cancer within 5 years of a COPD diagnosis (Fig. 2).

## Discussion

Nearly 90% of individuals with lung cancer will suffer from dyspnea near the time of death [15]. With limited tools for alleviating dyspnea, optimizing the management of related comorbidities such as COPD is imperative. Evidence suggests that COPD is underdiagnosed in both the primary care and cancer settings [7, 17]. Using a large administrative database incorporating data from a universal public healthcare system, we sought to determine the frequency of COPD diagnosis in a lung cancer population, as well as to assess the use of spirometry in diagnosis.

Despite the association between COPD and lung cancer, we found variable use of spirometry in the lung cancer population. Patients with early stage lung cancer were much more likely to undergo spirometry testing (90.6%) compared to those with more advanced disease (54.4%). Presumably, the higher figure relates to surgical work-up in early stage disease. However, the lower use of spirometry in later stage disease may lead to undertreatment of COPD, thereby contributing to dyspnea and reduced quality of life.

At the time of diagnosis of lung cancer, a prior diagnosis of COPD was found in 34.9% of individuals, a figure not dissimilar to previous reports employing spirometry [6, 7]. In our study, individuals with COPD were younger at the time of lung cancer diagnosis and were more likely to be diagnosed with early stage disease, although the differences were modest. While a small (*n* = 80) retrospective study also found that patients diagnosed with COPD prior to a diagnosis of cancer were more likely to have earlier stage disease, they found these individuals tended to be older [18]. The works of Mourante-Roibas and Zhang did not appreciate age or stage differences by COPD diagnosis on multivariate analysis [6, 7]. While a pre-existing diagnosis of COPD might intuitively be associated with more frequent imaging and earlier diagnosis of lung cancer, the data are not sufficiently clear to draw conclusions.

Outside of the specific context of lung cancer, tobacco use remains higher in rural than urban areas in North America [19, 20], and is inversely associated with socioeconomic advantage [21, 22]. Conversely, while global estimates of COPD suggest a higher urban prevalence [1], data from the USA and Ontario, Canada suggest a rural dominance [23, 24]. Our findings of greater rural prevalence of COPD among individuals diagnosed with lung cancer are consistent with these data.

Several factors may influence diagnosis of both COPD in the rural versus urban context, including access to medical staff and diagnostic equipment and cultural factors. The availability of primary care physicians and pulmonologists is less in rural US settings [25]. Perhaps accordingly, Fernadez-Villar et al found that fewer rural diagnoses of COPD were based upon an obstructive pattern on spirometry (Hazard Ratio 1.63) [26]. Qualitative work from Ontario suggests that while distance poses a barrier to the care of chronic diseases, culturally relevant teaching and support is also likely to be absent in rural locales. Furthermore, a culture of self-reliance may disincline individuals towards care [27]. It is possible that underdiagnosis of COPD may be greater in the rural setting.

In our study, patients with COPD and lung cancer had lower morbidity scores than patients with lung cancer alone. This differs from findings from Mourante-Roibas and colleagues who found that patients with lung cancer and COPD diagnosed by spirometry had higher Charlson scores than patients with lung cancer alone [6]. The difference may be that their diagnosis of COPD is prospective and based on spirometric data, whereas ours is based on physician claim and hospitalization data. Other data support the presence of concomitant illness in patients with COPD, with one health maintenance organization finding an average of 3.7 chronic comorbidities among individuals with COPD compared to 1.8 comorbidities in controls [28]. It is not clear whether difference in databases or differences in practice patterns are responsible for this discrepancy. One hypothesis is that comorbid conditions are being underdiagnosed and symptoms are instead attributed to their COPD, but this remains to be proven.

Dyspnea in lung cancer may have many causes, including endobronchial obstruction, effusions, thrombosis, infections, cardiac disease, and treatment toxicity. Thus, a diagnosis of COPD may be just one component of dyspnea in a given patient. In addition, not all patients are able to undergo spirometry for diagnosis, whether due to severe lung dysfunction, cough, strength, or pain issues. It should also be stated that cancer treatment has not definitively been shown to improve with COPD optimization. However, treated patients of all cancer stages with moderate or worse COPD may derive some relief of symptoms and modestly improved quality of life, as well as decreased frequency of hospitalization [10]. Given the limited palliative interventions for dyspnea from lung cancer, it is incumbent upon the clinician to seek all avenues to mitigate suffering.

The primary limitation of this study is that it is conducted using administrative data where incomplete or missing data is expected. For example, staging data was less complete in the earlier study period, meaning that just over 41% of patients were stage registered. Similarly, although the definition of COPD in our study is validated [14], it is reliant on physician billing or prior hospitalization for COPD rather than using spirometric data. Also, the presence of spirometry testing does not mean patients necessarily have COPD, since the presence of airflow obstruction is not recorded in the data we used. Finally, the ICES dataset is derived from the full population of the universally publicly funded Ontario healthcare system (> 13 million in 2014), and allows large scale comparison of reported diagnosis (COPD) as compared with actual use of testing (spirometry).

## Conclusions

In summary, using a large administrative database, we determined that spirometry is underutilized in the diagnosis of COPD, and that this remains a significant issue in patients diagnosed with lung cancer. Despite greater expected respiratory symptoms, spirometry was used less frequently in advanced stage disease. Increased use of spirometry may increase the diagnosis COPD in advanced stage lung cancer, allowing for improved dyspnea management and improved quality of life. Earlier diagnosis of COPD may also have important clinical implications for lung cancer diagnosis, given our findings of earlier stage diagnosis in the COPD population. Even as we develop more advanced oncologic therapies, our results argue for greater use of a simple, existing diagnostic maneuver.

## Data Availability

The dataset is unavailable as all ICES data is anonymous and kept on secured ICES servers, with analysis conducted through a virtual environment.

## Abbreviations

COPD: Chronic obstructive pulmonary disease
ICD: International Statistical Classification of Diseases and Related Health Problems RUB: resource utilization band
GOLD: Global Initiative for Chronic Obstructive Lung Disease
CCO: Cancer Care Ontario
